# High-dose interleukin-2 (hd-IL2) in treatment-naïve metastatic renal cell carcinoma (mrcc) - a 10 year single-site experience and outcome of prospective pathology-based patient selection

**DOI:** 10.1186/2051-1426-2-S3-P89

**Published:** 2014-11-06

**Authors:** Shien Chow, Manon Evans, Victoria Galvis, Rebecca Leach, Elizabeth Keene, Kevin Chan, Andrea Spencer-Shaw, Shanks Jonathan, Alaaeldin Shabalak, Fiona Thistlethwaite, Robert Hawkins

**Affiliations:** 1 Department of Medical Oncology, Christie NHS Foundation Trust, Manchester, Manchester, United Kingdom; 2 Department of Histopathology, Christie NHS Foundation Trust, Manchester, United Kingdom

## Background

Despite the advent of targeted therapies, mRCC remains difficult to treat with limited prospect of cure. HD-IL2 is the only agent consistently capable of achieving durable long-term remissions. Shablak et al have previously reported improved outcomes in patients prospectively stratified by a pathology-based criterion compared to an unselected population. We now present updated outcomes of mRCC patients given first-line treatment with HD-IL2 over a 10 year period from July 2003 to July 2013.

## Methods

The sample population consisted of 2 Cohorts: Cohort A (treated July 2003 - August 2006) where patients were retrospectively stratified for analysis by pathology into 'Favourable' and 'Other' groups ('Favourable' = < 10% papillary features, and at least 1 of > 50% alveolar/solid, or > 50% clear cell features); and Cohort B (treated August 2006 - July 2013) where patients were prospectively stratified. HD-IL2 was administered as per standard dosing and schedule. Clinical records were reviewed retrospectively; survivals were analysed by Kaplan-Meir methods. Carbonic Anhydrase 9 positivity (CAIX+) by immunohistochemistry was reviewed and recorded but was not used as selection criterion.

## Results

145 patients were included in the analysis. Median age 56, 72.6% male gender. Overall, 87.6% and 12.4% of patients had 'Favourable' and 'Other' pathology type respectively. Outcomes in patients with 'Favourable' histology were consistent across all time periods with overall response rates (ORR) and complete response (CR) rate being 41%,24%; 48%,23%; 47%,23% respectively in Cohort A, Cohort B and the total cohort.

Median overall survival (OS) of the total cohort was 55 months. Median OS for the HD-IL2 induced CR patients is not yet reached with 90% remaining alive and disease-free. A further 7 patients with 'Favourable' pathology achieved CR following surgical resection of residual disease with 4 currently alive and disease-free. Those with 'Other' pathology clearly responded less well.

We observed a high response rate (ORR 48%, CR 26%) in patients with CAIX+ >60%. 5 additional patients achieving CR were captured by lowering CAIX threshold from 80% to 60% positivity.

## Conclusions

Our study confirms that HD-IL2 remains an effective first-line treatment in well-selected patients with mRCC, emphasising further the sustained responses and long-term survival of those achieving a CR. Prospective application of our pathology-based selection criterion continues to correlate significantly with improved ORR and CR rates. CAIX appears predictive of response even at 60% (80% previously reported as significant), but should be analysed further prior to being considered as a selection adjunct.

